# Smartphone-Based Experience Sampling in People With Mild Cognitive Impairment: Feasibility and Usability Study

**DOI:** 10.2196/19852

**Published:** 2020-10-16

**Authors:** Sara Laureen Bartels, Rosalia J M van Knippenberg, Camilla Malinowsky, Frans R J Verhey, Marjolein E de Vugt

**Affiliations:** 1 Department of Psychiatry and Neuropsychology School for Mental Health and Neuroscience, Alzheimer Centre Limburg Maastricht University Maastricht Netherlands; 2 Division of Occupational Therapy Department of Neurobiology, Care Science and Society Karolinska Institutet Stockholm Sweden

**Keywords:** experience sampling method, mild cognitive impairment, cognition, feasibility, smartphones

## Abstract

**Background:**

Daily functioning of people with cognitive disorders such as mild cognitive impairment (MCI) is usually depicted by retrospective questionnaires, which can be memory-biased and neglect fluctuations over time or contexts.

**Objective:**

This study examines the feasibility and usability of applying the experience sampling method (ESM) in people with MCI to provide a detailed and dynamic picture of behavioral, emotional, and cognitive patterns in everyday life.

**Methods:**

For 6 consecutive days, 21 people with MCI used an ESM app on their smartphones. At 8 semi-random timepoints per day, participants filled in momentary questionnaires on mood, activities, social context, and subjective cognitive complaints. Feasibility was determined through self-reports and observable human-technology interactions. Usability was demonstrated on an individual and group level.

**Results:**

Of the 21 participants, 3 dropped out due to forgetting to carry their smartphones or forgetting the study instructions. In the remaining 18 individuals, the compliance rate was high, at 78.7%. Participants reported that momentary questions reflected their daily experiences well. Of the 18 participants, 13 (72%) experienced the increase in awareness of their own memory functions as pleasant or neutral.

**Conclusions:**

Support was found for the general feasibility of smartphone-based experience sampling in people with MCI. However, many older adults with MCI are currently not in possession of smartphones, and study adherence seems challenging for a minority of individuals. Momentary data can increase the insights into daily patterns and may guide the person-tailored development of self-management strategies in clinical settings.

## Introduction

Clinical questionnaires are commonly retrospective in nature and are thus potentially affected by a memory bias and thought to have low ecological validity [1]. As already cognitively healthy individuals over or underestimate past emotions and situations [2], this method might distort reality even more when people experience cognitive deficits. Moreover, within- or between-day fluctuations of health aspects are rarely taken into account, even though emotions and well-being vary depending on daily circumstances [3,4].

Momentary data collection, known as the experience sampling method (ESM) [5] or ecological momentary assessment [6], may offer a solution to this problem. The ESM uses diaries to gather information on symptoms, mood, activities, or social interactions in the moment they occur. Individuals fill in short questionnaires about current emotions and behaviors repeatedly over several days, which results in a high ecological validity and offers detailed insight into dynamic patterns [7]. Originally, ESM questionnaires had a paper-pencil format, but more recently, mobile devices such as smartphone apps have prevailed. Compared to paper-pencil diaries, technology-based ESM questionnaires can be filled in faster, reducing time burden and providing more details about the exact assessment time. Using the ESM, especially in combination with personalized feedback from a health care professional, increases awareness of and engagement in a healthy lifestyle and thus supports self-management [8,9]. The term self-management can be defined as “the individual's ability to manage their symptoms, treatment, physical and psychosocial consequences, and lifestyle changes inherent in living with a chronic condition” [10] and is a necessary skill to improve or maintain daily functioning.

A recent review reported that technology-based self-monitoring such as the ESM is already applied in various populations, including people with depression, chronic pain, or other health issues, to study behaviors and promote health [9]. In cognitively healthy older adults, momentary data collection seems feasible and acceptable [11] and is promising in individuals with brain injury [12] and after stroke [13].

One group of individuals that might also benefit from this diary approach are people with mild cognitive impairment (MCI). By definition, MCI is not thought to impact daily functioning greatly [14]; however, even small cognitive alterations can lead to changes in feelings, behaviors, self-perception, and social interactions [15]. Thus, self-management can be impaired when living with MCI.

To our knowledge, using the ESM in people with mild cognitive impairment (MCI) is rare. Daily or weekly paper-pencil diaries have been used to study momentary stressors and affect in MCI samples [16,17], but we are not aware of technology-based ESM studies in this population. Assessing the general feasibility of an unfamiliar and technology-based method is necessary, as people with MCI are commonly older and have amnestic deficits. Thus, individuals with MCI may find it challenging to process new information or handle unfamiliar technologies. Research shows, for example, that people with MCI find it more challenging to use everyday technology than older adults without cognitive impairments [18,19], which might also impact the feasibility of smartphone-based ESM. If feasible, applying ESM in people with MCI may reveal valuable insight into daily patterns of their lives that traditional assessments have been unable to depict. Furthermore, the ESM may promote awareness and self-management in this population, thus ultimately contributing to maintained or improved well-being.

This study aims to determine the feasibility and usability of smartphone-based experience sampling in people with MCI. An ESM app was installed on participants' smartphones and programmed with a high sampling frequency to capture various intra-individual states (ie, mood, subjective cognitive problems) and situations (ie, activities, social context). Self-reports of using the ESM and observations of the direct human-technology interaction were conducted as part of the feasibility assessment. Human-technology interaction refers here to the person's ability to manage the ESM smartphone app, including specific performance skills, environmental characteristics, and individual capacities.

The usability of momentary data was studied on an individual and group level, focusing on subjective cognition, daily activities, and stress experienced in relation to those activities. Studying the data on a group level can provide valuable information on the daily functioning of the MCI population in general, while individual data can illustrate within-person fluctuations. This may result in helpful person-tailored insights that not only foster individualized therapy but also the diagnostic process [20] and the monitoring of early changes in cognitive or behavioral alterations in MCI.

## Methods

### Sample

Participants were recruited from the memory clinic at the Maastricht University Medical Center (UMC) from June 2018 to January 2020. Inclusion criteria were (1) having a clinical diagnosis of MCI, according to Albert et al [14], (2) owning a smartphone with an Android or iOS operational system, (3) providing written informed consent, and (4) receiving written informed consent from a relevant other (ie, partner, family member, close friend) that was selected by the person with MCI and was recruited. Exclusion criteria were (1) insufficient abilities to participate in research (eg, a self-reported or relevant other–reported inability or lack of confidence to use a smartphone or to learn and remember the purpose of the study) and (2) severe health problems, such as a diagnosis of a somatic, psychiatric, or neurological disorder causing additional cognitive dysfunction. Both exclusion criteria were based on the clinical judgment of a psychologist or psychiatrist during the recruitment phase (eg, telephone conversations with a potential participant or relevant other).

The Medical Ethical Committee from the Maastricht academic hospital (azM) and Maastricht University approved the study (NL64310.068.17 / METC173055), and the protocol is registered on ToetsingOnline (64310). The authors comply with the Helsinki Declaration of 1975, as revised in 2008. All participants, including people with MCI and their relevant others, provided written informed consent before study participation.

### Experience Sampling Smartphone App

The PsyMate smartphone app [21] is a cloud-based platform developed at Maastricht University and Maastricht UMC (Multimedia Appendix 1). It is a tool for repeated momentary assessments in daily life that has been extensively studied and refined in mental health care [22]. In this study, the PsyMate was programmed to prompt participants 8 times a day over 6 consecutive days with an auditory and visual signal (“beep”) to fill in a short momentary assessment. A high sampling frequency of 8-10 beeps per day was thought to provide sufficient insight into various daily contexts while not disrupting the flow of everyday life. The duration of 6 days was chosen to capture both weekdays and weekend days. This set-up was based on previous feasibility studies [23,24]. Beeps occurred unpredictably in semirandom time blocks of 112.5 minutes between 7:30 AM and 10:30 PM and were available to be filled in for 15 minutes after the beep. In total, 27 ESM items were included and could be answered on a 7-point Likert scale or in a multiple-choice set-up (Multimedia Appendix 2) assessing mood (eg, “I feel cheerful”), physical well-being (eg, “I feel tired”), subjective cognition (eg, “Since the last beep, I had memory problems”), and context (eg, “Where am I?”). Participants classified their responses individually, meaning that “work,” for example, could mean paid employment for one individual while another individual selected this option for gardening or doing chores. A morning and evening questionnaire was also part of the ESM, asking the participant to reflect on the previous night (eg, “I slept well”) and day (eg, “Generally, I felt tense today”). These questionnaires were not prompted via beeps but were available during the morning and evening, to be filled in self-reliantly, and this data was not included in this study (Multimedia Appendix 3). The development of this questionnaire was based on previous ESM studies [22,25,26]. Questions on subjective cognition were added after consulting with ESM experts and clinicians (ie, psychologists, psychiatrists, and neuropsychologists from the UMC).

### Procedure

Participants were approached via the Alzheimer Center Limburg research database, consisting of patients with cognitive impairments who had previously expressed interest in being contacted for research purposes and had been previously recruited through UMC or by their treating health care professional at the memory clinic. A member of the research team called potential participants, checked general eligibility, verbally explained the study, and sent out information sheets. Participants were called by phone 1 week later, and if willing to participate, a date for the orientation session was set. A standardized protocol was used: (1) an orientation session, (2) an ESM training session, (3) a 6-day ESM period, and (4) a debriefing session. Only the person with MCI participated in the ESM training, the ESM period, and the debriefing session, but both the person with MCI and their relevant other were present at the orientation session. Sessions took place either at the hospital or at the participant's home, depending on the participant's preference. Participants could drop out at any time without providing a reason.

#### Orientation Session

After the study procedure was explained once more and final questions were clarified, informed consent was signed by the person with MCI and their relevant other. Next, sociodemographic information was collected and questionnaires were filled in assessing characteristics of the person with MCI either with self- or proxy-reports. At the end of this session, a date for the ESM training session was set. The ESM training was not combined with the orientation session so as not to overburden participants (as filling in a range of questionnaires can potentially be intense, confronting, and tiring). Thereby, we hoped to prevent participants from forgetting the ESM-training instructions due to information overload.

#### ESM Training Session

During the 30-minute training session, the PsyMate app was installed on the participant's smartphone, and the participant was instructed on how to respond to beeps, operate the app, and interpret the momentary questions. An example ESM questionnaire was filled in to familiarize participants with the procedure. The management of the app was observed, guided by the Management of Everyday Technology Assessment (META), to get a detailed picture of the human-technology interaction and performance skills [27]. All participants were briefed individually. A leaflet containing all instructions and contact information was handed out.

#### ESM Period

The PsyMate started sending beeps from the moment of installation; participants could respond on this day to train for filling in the momentary assessments, but they were instructed that the official 6-day ESM period would start the following day. On the second ESM day, a researcher called to check-in and solve potential technical problems or provide clarification.

#### Debriefing Session

This session took place 1 day after the last day of the ESM period. Participants were asked to report their general experiences using the app, and they received travel reimbursements and a small gift after participation but no financial reward.

### Instruments

#### Sociodemographic and Descriptive Information

Next to the sociodemographic information of the person with MCI (age, sex, education, living situation, years since first symptoms) and their relevant other (age, sex, relationship to person with MCI), reliable and valid instruments were filled in with the purpose of describing the sample. The Mini–Mental State Examination (MMSE) provided information on cognitive functioning [28]. If the MMSE had been administered by a health care professional at the memory clinic in the past 3 months, these scores were used to reduce the burden. Otherwise, the MMSE was part of the orientation session. Furthermore, the Guidelines for the Rating of Awareness Deficits (GRAD) were included as a semistructured interview to assess the degree of awareness of one’s own cognitive problems [29]; the GRAD compares the patient's information and the relevant other's view on the patient's history. Impaired awareness is defined as the absence of knowledge recognition of cognitive deficits and its impact [29]. The Hospital and Anxiety Depression Scale (HADS) was included to generate scores for generalized anxiety and depression [30,31], and the Perceived Stress Scale (PSS) measured the perception of stress [32]. The relevant other filled in the Neuropsychiatric Inventory Questionnaire (NPI-Q) for information on a variety of neuropsychiatric symptoms [33,34] as well as the Amsterdam Instrumental Activities of Daily Living (Amsterdam IADL), which specifically measures problems in instrumental activities in individuals with mild cognitive problems [35].

#### Feasibility Assessment: Self-Report

The feasibility was determined through the compliance rate of the ESM assessments and was regarded as satisfactory when >70% of the momentary questionnaires were filled in [12,23]. The subjective experience of using the ESM tool was assessed during the debriefing session through a semistructured interview, including ratings of the difficulty, time burden, interference with daily activities, and overall acceptability of the methodology. This interview followed a standardized protocol and included questions such as “Was this a normal week?” or “Did the PsyMate app hinder your daily occupations?” which were discussed and then rated by the participant on a 7-point Likert scale or categorically.

#### Feasibility Assessment: Observations

The Management of Everyday Technology Assessment (META) [36] was used during the ESM training session. This tool aims to identify the ability to manage technology among older adults with and without cognitive impairments by observing the direct human-technology interaction. The META consists of 4 parts, assessing (1) observable performance skills, (2) environmental characteristics, (3) the person's capacity, and (4) the perceived importance of the used technology. The fourth part (the perceived importance of the technology), as well as general information about the technology (eg, years of possession, amount of use), is answered by the individual via an interview. The first 3 parts are scored by the investigator on a 4-point scale: 4=competent handling and management (ie, no deficits in this skill disturbs or hinders the person's use of the technology; no difficulty); 3=deficits in this skill occasionally or slightly disturb the person's use of the technology (minor difficulty/problems); 2=deficits in this skill obviously disturb the person's use of the technology (major difficulty/problem); 1= deficits in this skill hinder the person's use of the technology, or the person is in need of assistance to perform the skill competently. For the first part (observable performance skills), 6 out of 11 performance skills were selected and scored, as the other 5 were not part of using a smartphone app (eg, coordinate different physical parts of the technology).

### Statistical Analyses

Descriptive analyses were conducted to summarize the sociodemographic information and background questionnaire scores. The compliance rate of the ESM day questionnaires, responses to the debriefing questionnaires, and META scores of the human-technology interaction were also analyzed using descriptive statistics. For the usability demonstration, only participants who filled in at least 30% of the ESM assessments were included, as a sufficient amount of information needs to be available to describe daily patterns [37]. Thus, momentary ESM data from dropouts collected via the PsyMate were not included in this part of the analysis. The momentary data were demonstrated on a group level using mood, context, feelings of tiredness, and subjective cognition items, and analyzed with descriptive statistics to assess the usability in this population in general [12]. The variable positive affect (PA) consisted of the ESM items “I feel cheerful,” “I feel energetic,” “I feel relaxed,” “I feel satisfied,” and “I feel enthusiastic.” The negative affect (NA) included “I feel down,” “I feel insecure,” “I feel irritated,” “I feel lonely,” “I feel anxious,” and “I feel guilty.” To demonstrate elements of daily functioning, an activity-related stress (ARS) variable was generated using “I can do this well” (reversed), “This requires effort from me,” and “I would rather do something else.” The ESM data collected with the PsyMate has a multilevel structure with beeps (level 1) nested within participants (level 2). Average scores of PA, NA, and ARS were thus person-mean centered to take the within-person effect into account. Factor analyses were conducted to ensure sufficient internal validity (Cronbach α=.86, .84, and .68 for the PA, NA, and ARS, respectively). PA, NA, and ARS were average, on a person-mean level. On an exemplary level, the subjectively experienced cognitive problems of 3 participants were demonstrated with line graphs over the course of the ESM period. The 3 participants were selected without specific criteria but with the aim of showing variation, giving a general impression of the ESM data, and demonstrating how the data can be used in clinical practice prospectively. Daily functioning using ARS was also demonstrated on an individual level by using data from 3 participants exemplary. Stata statistical software (version 13.0; StataCorp) was used for statistical analyses, and Excel (version 16.16.19; Microsoft) was used to create graphic visualizations.

### Data Availability Statement

The data is stored at Maastricht University. Due to ethical and legal regulations, the data is only accessible for the MUMC+ research team. Sharing data with another research team needs to be approved by the Medical Research Ethics Committee azM/UM, or participants need to sign a new informed consent sheet.

## Results

### Group Characteristics

**Figure 1:**
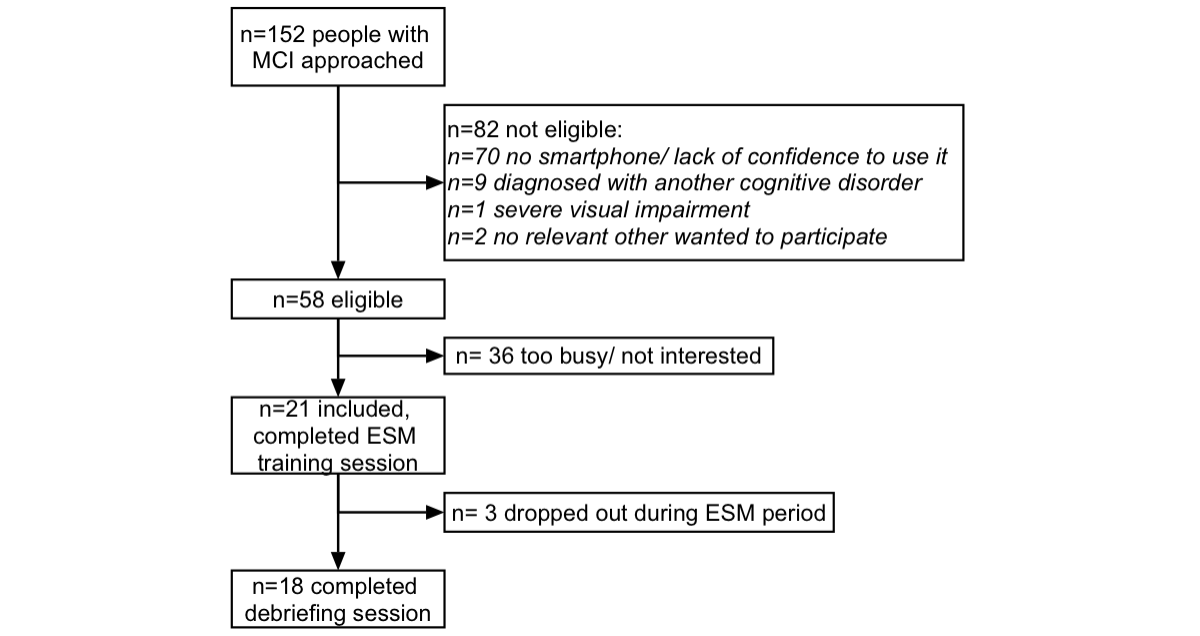
Flow chart of recruited participants with mild cognitive impairment (MCI). ESM: experience sampling method.

A total of 152 people with MCI were approached to participate in this study; 21 people with MCI signed informed consent. The participant flow is illustrated in Figure 1. Their relevant others also agreed to participate; the relevant others of the study participants had a mean age of 63.3 (SD 8.9, range 47-78) years, 6 were men and 15 were women, and 19 were the partners of the participants while 1 was a sibling and 1 was a friend. Table 1 provides an overview of the characteristics of the total sample. Multimedia Appendix 4 shows the details of the study completers and dropouts.

**Table 1:** Descriptive information about the participants with mild cognitive impairment (n=21).

Participant characteristics		Values
Age in years, mean (SD; range)		66 (7.1; 48-79)
**Sex, n (%)**		
	Men	16 (76)
	Women	5 (24)
**Level of education, n (%)**		
	Low (<9 years)	2 (10)
	Middle (9-10 years)	11 (52)
	High (>10 years)	8 (38)
**Employment status, n (%)**		
	Retired	14 (67)
	Working	3 (14)
	Unemployed	4 (19)
**Living situation, n (%)**		
	With partner	17 (81)
	With partner and children	1 (5)
	Alone	3 (14)
Years since first symptoms, mean (SD; range)		4.8 (4.0; 1-19)
Cognition (MMSE^a^), mean (SD; range)		28 (1.26; 27-30)
**Awareness (GRAD^b^), mean (SD; range)**		3.4 (0.67; 2-4)
	4: Intact, n (%)	10 (48)
	3: Mildly disturbed, n (%)	9 (43)
	2: Moderately disturbed, n (%)	2 (10)
	1: Absent, n (%)	—
Anxiety (HADS-A^c^), mean (SD; range)		11.8 (2.2; 6-15)
Depression (HADS-D^d^), mean (SD; range)		9.6 (1.4; 7-12)
Perceived stress (PSS^e^), mean (SD; range)		19.1 (4.5; 9-28)
Neuropsychiatric symptoms (NPI-Q^f^), mean (SD; range)		2.7 (2.1; 0-7)
Instrumental activities of daily living (IADL^g^), mean (SD; range)		57.2 (7.3; 45.9-69.9)

^a^MMSE: Mini–Mental State Evaluation; MMSE score range: 0-30, with higher scores indicating less cognitive difficulties.

^b^GRAD: Guidelines for the Rating of Awareness Deficits.

^c^HADS-A: Hospital and Anxiety Depression Scale–Anxiety; HADS scores range: 0-21 per scale (<7 noncases, 8-10 doubtful-cases, >11 definitive cases).

^d^HADS-D: Hospital and Anxiety Depression Scale–Depression; HADS scores range: 0-21 per scale (<7 noncases, 8-10 doubtful-cases, >11 definitive cases).

^e^PSS: Perceived Stress Scale; PSS scores range: 0-40, with higher scores indicating higher stress levels.

^f^NPI-Q: Neuropsychiatric Inventory Questionnaire; NPI-Q scores range: 0-36, with higher scores indicating a greater amount of neuropsychiatric behavior in the past month.

^g^IADL: Instrumental Activities of Daily Living; IADL *t* scores range: 20-80, with higher scores indicating better functioning; mean score=50 at memory clinics.

### Dropouts, Compliance, and Self-Report

In the study, 21 individuals started the ESM period, resulting in 673 beep records; 3 participants had problems using the ESM and did not complete the ESM period. These 3 dropouts had been eager to learn the app during the training session and their MMSE, other questionnaire scores, and general impression did not deviate outstandingly from the other participants. A statistical comparison between study completers and dropouts was not performed due to the small sample size.

The reasons for dropout were the following: Person A had problems using the right force pressing app buttons during the training session, forgot hearing aids repeatedly (according to partner) and thus did not react to the beeps, did not carry the smartphone along at all times, forgot the appointment, and seemed to generally deny cognitive problems. Person B expressed being very busy, only heard “some beeps” (no hearing problems, technical problems are unlikely according to IT specialist, reason unclear), and forgot the appointment for the debriefing session. Person C seemed generally nervous during the ESM training session (while expressing strong interest to participate), required very detailed and simple explanations of app use, and had forgotten instructions when contacted the following day. These 3 participants had not filled in the required 30% (16 beeps) to be included in the usability analysis, leading to a loss of 17 records (2.3%).

Of the 21 participants, 18 completed the ESM period and debriefing session, resulting in 656 valid beep records. On average, participants completed 38 beeps (SD 6.8; range 23-47) of the 48 beeps. The ESM compliance rate was 78.7%. Participants thought that the momentary questions reflected their experiences well (mean 4.83, SD 1.62) and that the PsyMate had little influence on their mood (mean 1.44, SD 1.15), activities (mean 1.61, SD 1.54), social interactions (mean 1.22, SD 0.73), or daily occupations (mean 1.39, SD 0.85). Filling in the momentary questions made participants marginally more aware of their activities (mean 2.17, SD 1.86) and moderately aware of their feelings (mean 3.56, SD 2.45) and memory (mean 4.56, SD 2.5). Of the 18 participants, 4 found increased awareness of their memory to be unpleasant, while 13 experienced it as pleasant or neutral. Table 2 provides detailed information on the general experience with the PsyMate and user-friendliness.

**Table 2:** General PsyMate app and user-friendliness evaluation [n=18; dropouts (n=3) were not included because they did not participate in the debriefing session].

General PsyMate app and user-friendliness evaluation			Scores (1=“not at all” – 7=“very much”)	
**General evaluation of PsyMate app, mean (SD; range)**				
	Was this a normal week?		5.06 (1.51; 2-7)	
	Did special events occur?		2.22 (1.73; 1-4)	
	Did the questions reflect your experiences well?		4.83 (1.62; 2-7)	
	Did the PsyMate app influence your mood?		1.44 (1.15; 1-5)	
	Did the PsyMate app influence your activities?		1.61 (1.54; 1-7)	
	Did the PsyMate app influence your social interactions?		1.22 (0.73; 1-4)	
	Did the PsyMate app hinder your daily occupations?		1.39 (0.85; 1-4)	
	Did you make mistakes when filling in the PsyMate app?		2.17 (0.92; 1-4)	
	**Did filling in the PsyMate app make you more aware of your feelings?**		3.56 (2.45; 1-7)	
		If so, did you experience this as pleasant? n^a^		7
		If so, did you experience this as neutral? n^a^		9
		If so, did you experience this as unpleasant? n^a^		1
	**Did filling in the PsyMate app make you more aware of your memory?**		4.56 (2.50; 1-7)	
		If so, did you experience this as pleasant? n^a,b^		6
		If so, did you experience this as neutral? n^a,b^		7
		If so, did you experience this as unpleasant?^a,b^		4
	**Did filling in the PsyMate app make you more aware of your activities?**		2.17 (1.86; 1-7)	
		If so, did you experience this as pleasant? n^a,b^		3
		If so, did you experience this as neutral? n^a,b^		14
		If so, did you experience this as unpleasant? n^a,b^		0
**Evaluation of PsyMate app user-friendliness, mean (SD; range)**				
	Were you able to read the text on the screen well?		6.06 (1.70; 1-7)	
	Could you hear the beep well?		6.44 (0.86; 4-7)	
	Did you have problems using the PsyMate app?		1.56 (1.65; 1-5)	
	Were the verbal explanations regarding the PsyMate app clear?		6.67 (0.60; 5-7)	
	Were the written explanations regarding the PsyMate app clear?		6.67 (0.60; 5-7)	
	Were the questions from the PsyMate app unclear or difficult?		2.28 (1.60; 1-7)	
	Did you experience the use of the PsyMate app burdensome with regard to the number of beeps?		1.44 (0.98; 1-5)	
	Did you experience the use of the PsyMate app burdensome with regard to length of one beep?		1.44 (0.62; 1-3)	
	Did you experience the use of the PsyMate app burdensome with regard to the sound?		2.33 ± 2.14 (1-7)	
	Did technical problems hinder you from filling in the beeps?^b^		1.88 1.09 (1-4)	

^a^Questions were not answered on a 7-point Likert scale but categorically.

^b^Missing response (n=1).

### Observation of the Human-Technology Interaction

The META revealed that most performance steps involved in using the PsyMate did not cause any difficulties (Table 3). However, using the appropriate force, tempo, and precision caused, on average, some disturbances (mean 3.48, SD 0.51). With regard to the environmental characteristics influencing the use of the PsyMate app during the training session, the contextual influence (ie, the presence of researchers, which could be potentially stressful) was observed as not hindering smartphone use (mean 3.9, SD 0.3; range 3-4), while the technological design (ie, screen and button size) was observed as somewhat disturbing (mean 3.38, SD 0.3; range 2-4). The overall judgment of the participants' capacity to use the app was reflected in the capacity to recall necessary information as not disturbing (mean 3.86, SD 0.36; range 3-4), just like the capacity to pay attention and focus (mean 3.81, SD 0.40; range 3-4) and the capacity to manage stress (mean 3.76, SD 0.45; range 3-4). Of the 21 participants, most participants (12/21) had had smartphones for more than 10 years; 5 had used a smartphone for 3-9 years, 1 had used it for 1-2 years, and 2 had it for less than 1 year (1 participant could not indicate the duration). All 21 participants experienced the technology as very important and not replaceable; 18 used their smartphones daily and 2 used it at least weekly (for the remaining 1 participant, there is a missing value).

**Table 3:** Assessment of observable performance skills when using the PsyMate app during the experience sampling method (ESM) training session (n=18).

Performance skill	Observation score^a^, mean (SD; range)
Identify service and function^b^	3.90 (0.31; 3-4)
Perform actions in logical sequence	3.95 (0.22; 3-4)
Manage series of number/letters^c^	4.0
Choose correct button or command	3.76 (0.45; 3-4)
Use appropriate force, tempo, and precision	3.48 (0.51; 3-4)
Identify information and respond adequately	3.95 (0.22; 3-4)

^a^Observation scores: range 1-4; 4=competent handling/management (ie, no deficits in this skill disturbs or hinders the person's use of the technology); 1=deficits in this skill hinder the person's use of the technology and/or the person is in need of assistance to perform the skill competently.

^b^n=1 missing, as skill was not observable.

^c^n=12 missing, as skill not observable.

### Usability of the Experience Sampling Data

#### Daily Patterns on a Group Level Over ESM Period

In general, the 18 participants experienced a high level of PA (mean 4.95, SD 0.66; range 3.94-6.13), a low level of NA (mean 1.95, SD 0.93; range 1.07-3.92) and a low to moderate level of ARS (mean 2.73, SD 0.74; range 1.71-4.05). They felt moderately tired (mean 3.64, SD 1.39; range 1-6.29) and had low to moderate problems with their memory (mean 3.01, SD 1.11; range 1.34-5.29), language (mean 2.04, SD 1.15; range 1-5.21), and concentration (mean 2.85, SD 1.36; range 1.05-4.96). With regard to their contextual patterns, participants spent most of their time at home (72%) (other locations: transport [9%]; at family's/friend's [5%]; at work [5%]; somewhere else [5%]; public place [4%]) engaging in household (22%) or relaxing activities (29%) (other activities: eating/drinking [10%]; something else [10%]; work [8%]; nothing [6%]; traveling [4%]; in conversation [4%]; self-care [4%]; sport/physical activity [3%]), and were often in company of their partner (45%) or alone (31%) (other social company: family [8%]; colleagues [4%]; co-occupants [4%]; friends [3%]; acquaintances [3%]; strangers [2%]).

#### Individual Profiles

**Figure 2:**
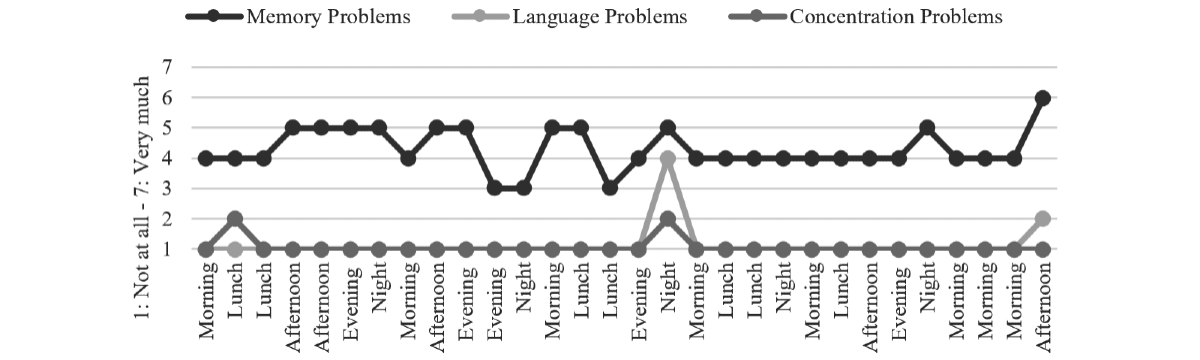
Person 1: subjectively experienced cognitive problems.

**Figure 3:**
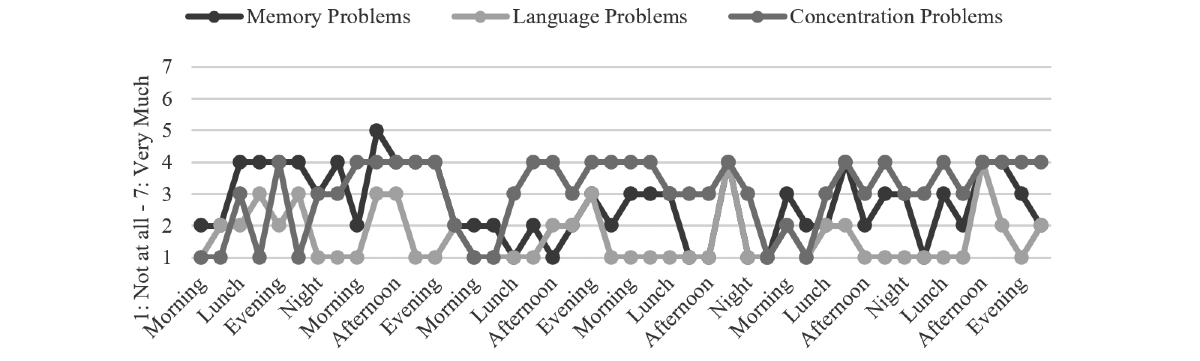
Person 2: subjectively experienced cognitive problems.

**Figure 4:**
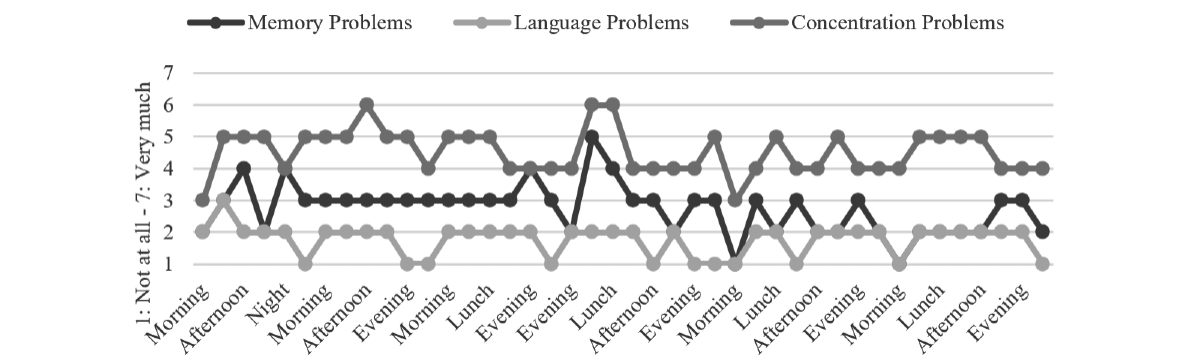
Patient 3: subjectively experienced cognitive problems.

To illustrate the variability that can be studied using momentary data, several descriptive examples are presented, focusing on the subjectively experienced cognitive problems, daily activities, and activity-related stress in everyday life. These participants were selected without specific criteria but with the aim of visually illustrating fluctuations within subjects, variables, and days (Figures 2-4). An unspecified heterogeneity is present, while no statistical differences within and between subjects were tested. Some suggestions for personalized feedback conversations between health care professionals and the individuals are provided as well.

As shown in Figure 2 and Table 4, Person 1 reports mainly moderate memory problems, while language and concentration abilities are overall subjectively unimpaired. Person 1 engages in doing “nothing” 24% of the time. This activity shows personally higher levels of ARS, while “relaxing” has lower levels of ARS. The person engages in “work” (note: not necessarily paid) 10% of the time, which also shows a personally higher level of ARS. When discussing this data, increased engagement in relaxation and coping with work could be targeted.

**Table 4:** Person 1: daily activities and levels of activity-related stress.

Daily activities	Daily time (%)	Activity-related stress level^a^
Nothing	24	3.14
Relaxing	7	1.78
In conversation	3	2
Something else	50	2
Eating/drinking	3	2
Work	10	3.67
Travel	3	2.07

^a^Scale: 1 (not at all) – 7 (very much). This data stands in relation to the fluctuations of cognition (Figure 2).

As shown in Figure 3 and Table 5, Person 2 reports cognitive problems that fluctuate across all 3 domains. Conversations (2% of activity engagement) seem to be the most stressful (personally higher level of ARS). In this case, dealing with cognitive problems and developing coping strategies for conversations might be useful for the individual.

**Table 5:** Person 2: daily activities and levels of activity-related stress.

Daily activities	Daily time (%)	Activity-related stress level^a^
Household	32	2.89
Relaxing	39	2.69
In conversation	2	3.33
Sports/physical activity	5	2.33
Eating/drinking	9	2.33
Work	2	2.67
Self-care	9	3.08
Traveling	2	2

^a^Scale: 1 (not at all) – 7 (very much). This data stands in relation to the fluctuations of cognition (Figure 3).

As shown in Figure 4 and Table 6, Person 3 has subjective cognitive impairments in all 3 domains that fluctuate somewhat simultaneously. On some mornings, the cognitive problems seem to be milder. Relaxation activities, which report low levels of ARS, are the main activity of Person 3 (48%). Nevertheless, doing nothing (12%) and working (7%) might be topics to discuss to optimize self-management.

**Table 6:** Person 3: daily activities and levels of activity-related stress.

Daily activities	Daily time (%)	Activity-related stress level^a^
Household	5	4.17
Relaxing	48	3.3
Something else	14	3.56
Sports/physical activity	5	3.5
Eating/drinking	2	4
Work	7	4.67
Self-care	7	3
Nothing	12	5.33

^a^Scale: 1 (not at all) – 7 (very much). This data stands in relation to the fluctuations of cognition (Figure 4).

## Discussion

### Principal Findings

This study evaluates the feasibility and demonstrates the usability of a smartphone-based ESM in people with MCI. Several important findings emerged: (1) in study completers, the compliance rate was high and subjective ratings of the ESM procedure were positive; (2) the observable human-technology interaction between participants and the ESM app was generally unproblematic; (3) raising awareness for one’s own cognitive problems through ESM can be unpleasant for some individuals; and (4) cognitive issues (eg, forgetfulness) may lead to the inability to use the ESM.

Previous research found that the compliance rate, also referred to as adherence, use, or engagement, to technology-based self-monitoring such as ESM lies between 51-86% in middle-aged and older adults [9,38]. Therefore, the reported 78.7% of completed assessments in this study is a strong indication for the feasibility of ESM in a majority (18/21, 85%) of our MCI sample. A high sampling frequency, which was applied in this study with 8 beeps per day, is not thought to hinder ESM use, while the length of the questionnaires can increase burden [39]. The overall positive participants’ feedback on the procedure, including the length and frequency, supports the chosen ESM set-up. Additionally, the human-technology interactions were observed as overall unproblematic. Occasionally, a participant's inappropriate tempo, force, or precision of clicking on app buttons disturbed usage slightly. As older adults may benefit from large buttons and screens without scroll functions [40], it would be advisable to rotate the screen, increase button sizes, or provide a touch-pen to ease the app use even further. In addition to dexterity, older adults might also have hearing issues. In this study, one of the dropouts did not wear their hearing aids, which might have contributed to their inability to use the ESM and discontinuation of the trial.

According to the social cognition theory, self-monitoring can raise awareness for one’s own emotions or behaviors [41]. Repeated momentary assessments can use this increased awareness to promote behavioral changes towards healthy lifestyles [22,42] as well as improve mental well-being [43]. Within this study, there was no intention to change daily patterns, but nevertheless, participants became more aware of their memory abilities through repeated self-assessments. Of the 18 participants, 13 individuals experienced this as pleasant or neutral, but 4 reported this to be unpleasant. Similar side-effects of the ESM have previously been reported, and a suggestion could be to use positive formulations in the ESM questions [44]. For example, instead of asking about cognitive problems (eg, “Since the last beep, I had memory problems”), abilities could be targeted instead (eg, “I can remember well”). In this study, the experience of using ESM was discussed during the debriefing session, and in 1 case, a participant was advised to consult a health care professional for further treatment for cognition-related stress. In clinical settings, treating health care professionals may discuss experiences and increased awareness to develop coping strategies [45]. The individual profiles section highlights topics that may be discussed on an individual level, such as activities that elicit low stress (eg, relaxation) or the potential need for assistance or new coping strategies. Studies suggest that those feedback conversations could focus on positive emotions to increase resilience to stress [46] and stimulate goal-directed behavior [47]. This kind of feedback has shown to improve well-being, for instance, in an ESM-based intervention for carers of people with dementia [26]. The ultimate goal when using ESM is to support self-management through increase awareness for one's own abilities and orientate attention towards positive and meaningful aspects of daily life.

A small number of participants were unable to complete the experience sampling period. In older adults with undiagnosed subjective cognitive concerns, nonadherence to momentary assessments is thought to be greatly influenced by cognitive issues [48]. In this study, predicting dropouts using standardized instruments such as the MMSE was impossible. A systematic review reports that the averaged MMSE score in MCI samples seems to range from 23.1 to 28.7 [19], indicating a great variability of cognitive abilities in this population and that participants of this study potentially had relatively mild MCI. However, the MMSE has a limited discrimination between cognitively health adults and people with MCI, and other tests with a higher sensitivity (eg, Hopkins Verbal Learning Test) could have been used to determine study eligibility [49]. All participants were eager to participate, while no clear indication for exclusion could be identified. Follow-up phone calls were helpful to notice difficulties early. Dropouts seem to blame their inability to participate on the technology (eg, “It did not beep”). Admitting problems with technology might be easier than admitting other cognitive deficits, as even young and healthy individuals may occasionally face difficulties with technology. Further, reduced illness insight and cognitive deficits could have influenced the ability to use the ESM. The latter is supported by reports from participants and relevant others, stating that smartphones or hearing aids were forgotten, thus interfering with the ESM use. To prevent injustice in health care, all individuals with MCI motivated to use the ESM should be given a chance to do so, and frustration can be prevented through follow-ups, close guidance, and open communication.

Generally, the ESM group data revealed subjective problems with memory, concentration, and language in everyday life. This finding is in line with traditional neuropsychological assessments reporting a variety of cognitive deficits in MCI, of which memory is commonly most dominant [50]. A moderate level of fatigue has also been found in a healthy sample using the ESM [24] and may thus not be directly related to the cognitive deficits. To determine significant differences from healthy older adults, a control group is prospectively necessary. Furthermore, associations between daily fatigue, context, mood, and cognitive problems experienced by individuals may be studied using multilevel analysis [12].

### Future Directions

On an individual level, cognitive fluctuations indicate trends of diversity both within and between subjects. The heterogeneity of the MCI group has been highlighted before [51], but this is one of the first studies to provide such a detailed insight into daily patterns using smartphone-based ESM. Next to the subjective evaluation of cognitive problems in everyday life, objective momentary cognition tasks can be added to this ESM app. The feasibility of 2 tasks has recently been tested in healthy individuals [52] and holds promise for future studies to describe a comprehensive picture of cognitive abilities. The ESM may also be useful to compare daily patterns of subjective or objective cognitive functioning in different neurological and psychiatric disorders.

Additionally, activity-related stress levels seem to vary between activities as working, for example, shows a trend for high levels of stress. This study is unfortunately not able to statistically explore activity patterns in people with MCI, but future research might follow up on this idea. Research shows that complex tasks are affected early on in the process of cognitive decline [53], and cognitive difficulties may decrease the ability of individuals with MCI to work [54]. Our understanding of necessary adjustments and ways to support working, particularly employment, when living with MCI are limited [55], but the insights gained from the participants in this study highlight the need to study working and employment as a potential stressor in this population further. High levels of anxiety and depression are commonly observed in MCI [56], as they were in this sample, and may also stand in relation to stress and cognitive deterioration [57]. As highlighted above, developing coping strategies and focusing on positive emotions might support daily well-being, including work-related stress. The ESM can be a useful tool to relate functional fluctuations with contexts and activities and thus understand patterns and networks in people with MCI, both on a within- and between-subject level [58].

### Limitations

Some critique regarding the ESM and study limitations need to be acknowledged. It is recommended not to overinterpret single items but rather to use momentary data as a starting point for a conversation about one's self-management and coping. Generally, many people (n=70) approached for the study had no smartphone or did not feel confident to participate in a smartphone-based study. This outcome indicates that there is a bias towards individuals with a higher technology familiarity to benefit from digital innovations in research and clinical work. Over the next decade, this bias might decrease, but researchers and clinicians need to be aware of this gap to not neglect individuals in need of support. Potentially, traditional paper-pencil diaries might be an alternative for people with MCI [16,17] that cannot or do not want to use smartphones. However, cognitive problems (eg, forgetting paper diary) or hearing problems (eg, not hearing the beeps from a prompting device) could still interfere. As learning and using a new technology is an intertwined process [59], and training is a key component for older adults to increase confidence and self-efficacy when using technology [60], prospective individuals with MCI who are not confident in their abilities to use a smartphone could receive training sessions and additional guidance. Unfortunately, this study is not able to determine if individuals with MCI would also be able to learn smartphone and ESM use together. Furthermore, the findings may be affected by a sex and education bias, as 76% (16/21) were men and only 10% (2/21) were low-educated. In addition, the etiology of MCI was not determined, resulting in an unspecified heterogeneity. As indicated by the MMSE, this MCI sample might have relatively mild cognitive problems, and a replication of our findings in a broader MCI sample might be necessary to increase the generalizability of the results. Detailed descriptive information about MCI subgroups could prospectively be added. The small sample size orientated on other feasibility studies [12,61] may limit the generalizability of the results, but the great number of assessments still result in a rich data set [22]. Finally, the study represents a specific group of people with MCI in possession of their own smartphones, and this recruitment criteria needs to be kept in mind when applying the ESM in future studies or clinical settings.

### Conclusion

Technology-based ESM can be a useful addition to clinical questionnaires to reveal detailed moment-to-moment fluctuations, contextual patterns, and individual differences in subjectively experienced cognitive problems, affect, and activities. This feasibility study is a relevant step to better understand and support people with MCI in their everyday lives. Momentary data may prospectively be used to study individual and group-based patterns in this population and develop person-tailored self-management strategies.

## Appendix

Multimedia Appendix 1 Interface of the experience sampling method (ESM) smartphone app "PsyMate.".

Multimedia Appendix 2 Experience sampling method (ESM) item list of the day questionnaire.

Multimedia Appendix 3 Morning and evening questionnaires.

Multimedia Appendix 4 Descriptive information of the study completers (n=18) and dropouts (n=3).

## Abbreviations

Amsterdam IADL: Amsterdam instrumental activities of daily living
ARS: activity-related stress
azM: academisch ziekenhuis Maastricht
ESM: experience sampling method
GRAD: Guidelines for the Rating of Awareness Deficits
HADS: Hospital and Anxiety Depression Scale
MCI: mild cognitive impairment
META: Management of Everyday Technology Assessment
MMSE: Mini–Mental State Examination
NA: negative affect
NPI-Q: Neuropsychiatric Inventory Questionnaire
PA: positive affect
PSS: perceived stress scale
UMC: Maastricht University Medical Center
