# Supplementary figures and images for: Smartphone-Based Experience Sampling in People With Mild Cognitive Impairment: Feasibility and Usability Study
Source: JMIR Aging. 2020 Oct 16;3(2):e19852. doi: 10.2196/19852 (PMC7600012; doi:10.2196/19852)

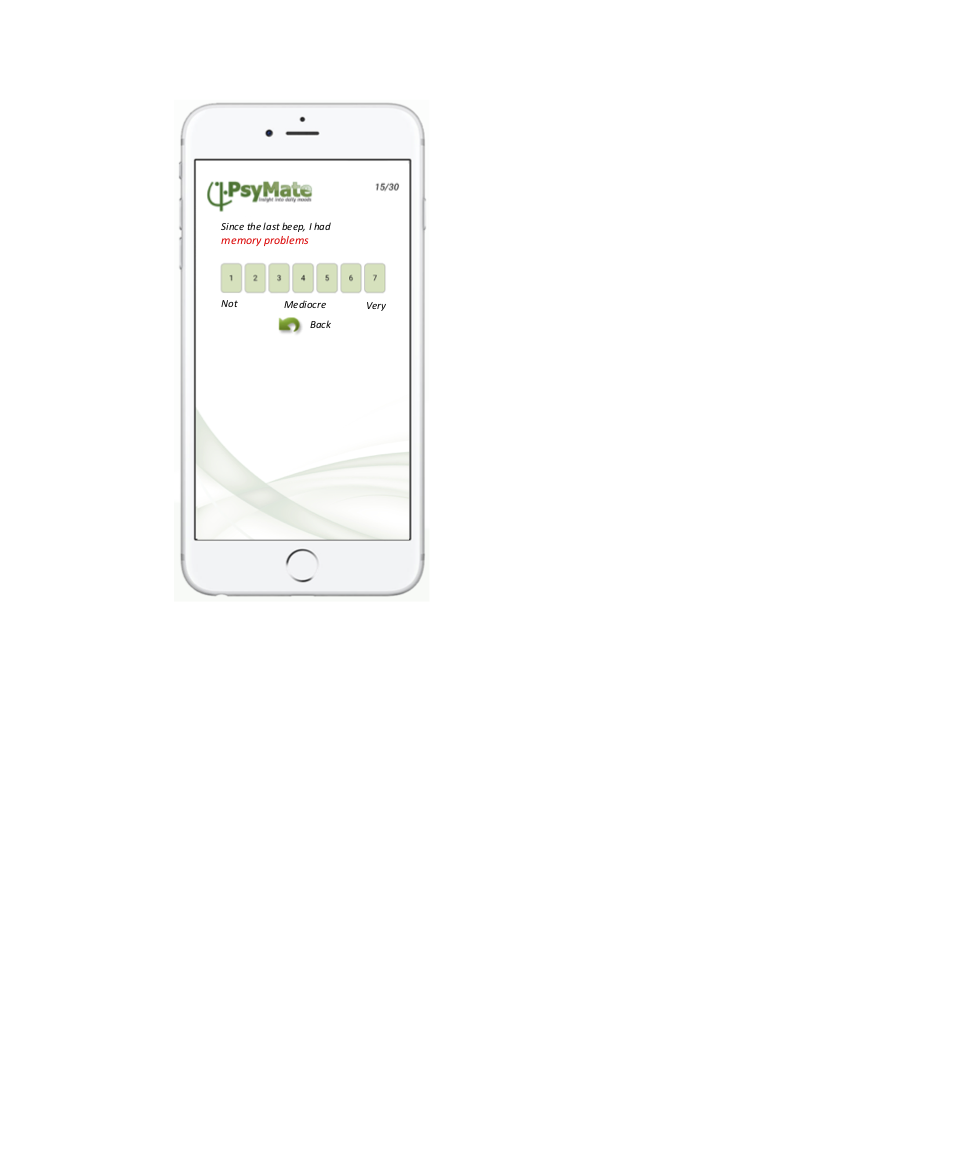

Supplement: Multimedia Appendix 1 [file aging_v3i2e19852_app1.png]
