# Supplementary material for: Smartphone-Based Experience Sampling in People With Mild Cognitive Impairment: Feasibility and Usability Study
Source: JMIR Aging. 2020 Oct 16;3(2):e19852. doi: 10.2196/19852 (PMC7600012; doi:10.2196/19852)
Supplement: Multimedia Appendix 2 [file aging_v3i2e19852_app2.docx]

| *Concept*/ ESM Items | Response options |
| --- | --- |
| *Mood*  I feel cheerful.  I feel energetic.  I feel insecure.  I feel relaxed.  I feel gloomy.  I feel irritated.  I feel satisfied.  I feel lonely.  I feel enthusiastic.  I feel anxious.  I feel guilty.  I worry. | 7-point scale (1 ‘not at all’ to 7 ‘very much’)  7-point scale (1 ‘not at all’ to 7 ‘very much’)  7-point scale (1 ‘not at all’ to 7 ‘very much’)  7-point scale (1 ‘not at all’ to 7 ‘very much’)  7-point scale (1 ‘not at all’ to 7 ‘very much’)  7-point scale (1 ‘not at all’ to 7 ‘very much’)  7-point scale (1 ‘not at all’ to 7 ‘very much’)  7-point scale (1 ‘not at all’ to 7 ‘very much’)  7-point scale (1 ‘not at all’ to 7 ‘very much’)  7-point scale (1 ‘not at all’ to 7 ‘very much’)  7-point scale (1 ‘not at all’ to 7 ‘very much’)  7-point scale (1 ‘not at all’ to 7 ‘very much’) |
| *Physical well-being*  I feel good.  I feel tired. | 7-point scale (1 ‘not at all’ to 7 ‘very much’)  7-point scale (1 ‘not at all’ to 7 ‘very much’) |
| *Subjective cognitive complaints*  Since the last ‘beep’, I had memory problems.  Since the last ‘beep’, I had speech problems.  Since the last ‘beep’, I had concentration problems. | 7-point scale (1 ‘not at all’ to 7 ‘very much’)  7-point scale (1 ‘not at all’ to 7 ‘very much’)  7-point scale (1 ‘not at all’ to 7 ‘very much’) |
| *Context: Activity*  What do I do?  I can do this well.  I would rather do something else.  This requires effort from me.  I am present with my thoughts. | Multiple-choice (Work; household; self-care; relaxation; sport, physical activity; eating, drinking; traveling, on the way; in a conversation; something else; nothing)  7-point scale (1 ‘not at all’ to 7 ‘very much’)  7-point scale (1 ‘not at all’ to 7 ‘very much’)  7-point scale (1 ‘not at all’ to 7 ‘very much’)  7-point scale (1 ‘not at all’ to 7 ‘very much’) |
| *Context: Location*  Where am I? | At home; at family’s/friend’s place; at work; health care setting; public space; transport; somewhere else |
| *Context: Social Company*  With whom am I?  Branching (in company/alone)  I like this company?/ being alone?  I would rather be alone?/ in company? | Partner; family; housemate; friend; colleague; acquaintance; stranger; nobody  7-point scale (1 ‘not at all’ to 7 ‘very much’)  7-point scale (1 ‘not at all’ to 7 ‘very much’) |
| *General*  This alert disturbed me. | 7-point scale (1 ‘not at all’ to 7 ‘very much’) |
