# Supplementary material for: Smartphone-Based Experience Sampling in People With Mild Cognitive Impairment: Feasibility and Usability Study
Source: JMIR Aging. 2020 Oct 16;3(2):e19852. doi: 10.2196/19852 (PMC7600012; doi:10.2196/19852)
Supplement: Multimedia Appendix 3 [file aging_v3i2e19852_app3.docx]

| Questionnaire | *Concept*/ ESM Items | Response options |
| --- | --- | --- |
| Morning Questionnaire | How long did it take me to fall asleep last night?  How many times did I wake up during the night?  How long was I awake this morning before I got up?  I slept well.  I feel well rested.  I am looking forward to this day.  Thank you! | 0-5 min.; 5-15min.; 15-30 min.; 30-45min.; 45 min – 1 h; 1-2 h; 2-4 h; >4 h  0; 1; 2; 3; 4; >5  0-5 min.; 5-15min.; 15-30 min.; 30-45min.; 45 min – 1 h; 1-2 h; 2-4 h; >4 h  7-point scale (1 ‘not at all’ to 7 ‘very much’)  7-point scale (1 ‘not at all’ to 7 ‘very much’)  7-point scale (1 ‘not at all’ to 7 ‘very much’) |
| Evening Questionnaire | Generally, I felt good today.  Generally, I felt tired today.  Generally, I felt tense today.  Generally, I felt like I could concentrate today.  Generally, I felt forgetful today.  Good night! | 7-point scale (1 ‘not at all’ to 7 ‘very much’)  7-point scale (1 ‘not at all’ to 7 ‘very much’)  7-point scale (1 ‘not at all’ to 7 ‘very much’)  7-point scale (1 ‘not at all’ to 7 ‘very much’)  7-point scale (1 ‘not at all’ to 7 ‘very much’) |
