# Supplementary material for: Smartphone-Based Experience Sampling in People With Mild Cognitive Impairment: Feasibility and Usability Study
Source: JMIR Aging. 2020 Oct 16;3(2):e19852. doi: 10.2196/19852 (PMC7600012; doi:10.2196/19852)
Supplement: Multimedia Appendix 4 [file aging_v3i2e19852_app4.docx]

| Variables | Study completers (n=18) | Dropouts (n=3) |
| --- | --- | --- |
| Age in years (M, SD, range) | 65 ± 6.9 (48-73) | 69 ± 9.5 (60-79) |
| Sex (n, % male) | 14 (78%) | 2 (66%) |
| Level of education (n, %)  Low (< 9 years)  Middle (9-10 years)  High (>10 years) | 1 (5.6%)  10 (55.56%)  7 (38.9%) | 1 (33.3%)  1 (33.3%)  1 (33.3%) |
| Employment status (n, %)  Retired  Working  Unemployed | 12 (66.6%)  2 (11.1%)  4 (22.2%) | 2 (66.6%)  1 (33.3%)  - |
| Living situation (n, %)  With partner  With partner and children  Alone | 14 (77.8%)  1 (5.6%)  3 (16.6%) | 3 (100%)  -  - |
| Years since first symptoms (M, SD, range) | 4.3 ± 3.9 (1-19) | 7.7 ± 4.0 (3-10) |
| Cognition; MMSE (M, SD, range) | 27.9 ± 1.2 (27-30) | 28.3 ± 1.5 (27-30) |
| Awareness; GRAD (M, SD, range)  4: Intact (n, %)  3: Mildly disturbed (n, %)  2: Moderately disturbed (n, %)  1: Absent (n, %) | 3.4 ± 0.7 (2-4)  9  7  2  - | 3.3 ± 0.6 (3-4)  1  2  -  - |
| Anxiety; HADS-A (M, SD, range)  Depression: HADS-D (M, SD, range) | 11.6 ± 2.3 (6-15)  9.7 ± 1.4 (7-12) | 13 ± 1.7 (11-14)  9.3 ± 1.2 (8-10) |
| Perceived stress; PSS (M, SD, range) | 19.1 ± 4.4 (9-28) | 14.7 ± 1.2 (14-16) |
| Neuropsychiatric symptoms; NPI-Q (M, SD, range) | 2.7 ± 2.2 (0-7) | 2.7 ± 2.1 (1-5) |
| Instrumental activities of daily living;  IADL (M, SD, range) | 57.0 ± 7.0 (45.9-69.9) | 58.2 ± 11 (48.1-69.9) |

*Note: MMSE score range: 0-30, with higher scores indicating less cognitive difficulties. HADS scores range: 0-21 per scale (<7 non-cases; 8-10 doubtful-cases; >11 definitive cases). PSS scores range:: 0-40, with higher scores indicating higher stress levels. NPI-Q scores range:0-36, with higher scores indicating greater amount of neuropsychiatric behavior in the past month. IADL t-scores range: 20-80, with higher scores indicating better functioning, 50=mean score at memory clinics.*
